# Enhanced Detection and Segmentation of Sit Phases in Patients with Parkinson’s Disease Using a Single SmartWatch and Random Forest Algorithms

**DOI:** 10.3390/s25196104

**Published:** 2025-10-03

**Authors:** Etienne Goubault, Camille Martin, Christian Duval, Jean-François Daneault, Patrick Boissy, Karina Lebel

**Affiliations:** 1 Institut de Recherche Robert-Sauvé en Santé et en Sécurité du Travail (IRSST), 505 Boul. de Maisonneuve O, Montréal, QC H3A 3C2, Canada; 2 Centre de Recherche sur le Vieillissement, CIUSSS de l’Estrie—CHUS, Sherbrooke, QC J1H 4C4, Canada; camille.martin3@usherbrooke.ca (C.M.); patrick.boissy@usherbrooke.ca (P.B.); karina.lebel@usherbrooke.ca (K.L.); 3 Département des Sciences de l’Activité Physique, Université du Québec à Montréal, Montréal, QC H2X 1Y4, Canada; duval.christian@uqam.ca; 4 Centre de Recherche de l’Institut Universitaire de Gériatrie de Montréal, Montréal, QC H3W 1W6, Canada; 5 Department of Rehabilitation and Movement Sciences, Rutgers University, Newark, NJ 07107, USA; jf.daneault@rutgers.edu; 6 Département de Chirurgie, Service d’Orthopédie, Faculté de Médecine et des Sciences de la Santé, Université de Sherbrooke, Sherbrooke, QC J1H 5H3, Canada; 7 Département de Génie Électrique et de Génie Informatique, Université de Sherbrooke, Sherbrooke, QC J1K 2R1, Canada

**Keywords:** Activities of daily living, inertial sensors, automatic detection, movement disorders, random forest

## Abstract

Background. Automatic detection of *Sit* phases in people with Parkinson’s disease (PD) using a single body-worn sensor is crucial for enhancing long-term, home-based monitoring of mobility. Aim. The aim of this study was to enhance the accuracy of detecting and segmenting *Sit* phases in people with PD using a single SmartWatch worn at the ankle. Method. Twenty-two patients living with PD performed activities of daily living that incorporate repeated transitions to a seated position in a simulated free-living environment during 3 min, 4 min, and 5 min trials. Tri-axial accelerations and angular velocities of the right or left ankle were recorded at 50 Hz using a SmartWatch. Random forest algorithms were trained using raw and filtered data to automatically detect and segment *Sit* phases. Sensibility, specificity, and F-scores were calculated based on manual segmentation using the OptiTrack motion capture system. Results. Sensibility, specificity, and F-score achieved 78.3%, 93.8%, and 84.7% for *Sit* phase detection of the 3 min trial; 78.8%, 85.5%, and 80.6% for *Sit* phase detection of the 4 min trial; and 71.6%, 84.8%, and 75.6% for *Sit* phase detection of the 5 min trial. The median time difference between the manual and automatic segmentation was 0.95s, 0.89s, and 0.84s, respectively, for the 3 min, 4 min, and 5 min trial. Conclusion. This study demonstrates that a random forest algorithm can accurately detect and segment *Sit* phases in people with PD using data from a single ankle-worn SmartWatch. The algorithm’s performance was comparable to manual segmentation, while substantially reducing the time and effort required. These findings represent a meaningful step forward in enabling efficient, long-term, and home-based monitoring of mobility and symptom progression in people with PD.

## 1. Introduction

People with Parkinson’s disease (PD) often experience limited mobility in their activities of daily living (ADLs), which can lead to loss of independence and a decrease in their quality of life. In recent years, increasing attention has been directed toward the use of commercially available body-worn sensors to assess patients’ mobility [1], with the aim of improving the management of PD symptoms through long-term, home-based monitoring [2].

Lower-back inertial sensors have been used to classify walking phases in patients with neurological conditions resulting from cerebral lesions, as well as in individuals who have undergone or are preparing for orthopaedic surgery, to support rehabilitation assessment [3]. They have also been used to detect gait events and extract mobility parameters in people with PD, providing insights into motor symptoms and functional status [2].

While lower-back sensors have demonstrated strong performance in capturing mobility-related signals, they are frequently reported as less comfortable compared to wrist- or ankle-worn devices, raising concerns about their acceptability for continuous, long-term monitoring in daily life. This limitation has driven growing interest in the use of inertial sensors that are embedded in SmartWatches, which offer a more user-friendly and socially acceptable alternative. When worn at the wrist or ankle, these devices can capture a range of gross motor activities that are central to evaluating mobility, including walking, turning, sitting down, and standing up. In healthy populations, motor activity detection with wrist-worn SmartWatches has often relied on arm swing recognition patterns or empirical thresholding techniques to differentiate between activity states [4]. These methods, however, may not generalize effectively to people with PD. Arm swing amplitude is typically reduced or asymmetric in people with PD, making algorithms that are designed for healthy controls less robust in this context [5]. Furthermore, PD-related motor complications such as tremor and dyskinesia—often localized in distal limb segments and fluctuating in intensity—introduce additional variability in wrist-based sensor signals [6,7]. These artifacts can obscure meaningful mobility patterns, complicating the reliable detection of gross motor activities.

To improve the likelihood of success in implementing wearable technologies for mobility and symptom monitoring in people with PD, tailored approaches that account for disease-specific motor characteristics are required. Building on this premise, we recently introduced algorithms designed for the automatic detection and segmentation of gross motor activities that are commonly performed during ADLs in people with PD [8]. These algorithms leverage data from a single SmartWatch worn at the ankle, a placement that offers several advantages over the more conventional wrist location. The ankle provides a more direct proxy of lower-limb movement, reducing the confounding effects of reduced arm swing, tremor, or dyskinesia that often compromise wrist-based measurements in people with PD. By focusing on activities such as walking, turning, sitting down, and standing up—movements that are both fundamental to daily life and highly sensitive to PD-related impairments—this approach aims to capture ecologically valid markers of mobility in naturalistic settings. Automated segmentation of these activities not only facilitates more accurate monitoring of motor fluctuations and functional capacity but also provides a foundation for integrating wearable-based assessments into clinical decision-making and personalized disease management strategies. Ultimately, this line of work underscores the potential of strategically positioned, single-device solutions to balance patient acceptability with the clinical precision that is needed for real-world deployment of digital health technologies in people with PD.

The proposed algorithms demonstrated strong performance in detecting and segmenting mobility-related activities in a simulated free-living environment. Specifically, *Walking* and *Turning* were recognized with high precision, achieving F1-scores of 96% and 92%, respectively. This robust performance can be explained by the use of ankle-mounted inertial data, which is largely unaffected by PD-related motor impairments such as reduced arm swing [5], tremor, and dyskinesia—factors that typically compromise wrist-based detection approaches. In contrast, the classification of transitional activities such as sitting down and standing up proved more challenging. The detection accuracy for these sit-to-stand phases was substantially lower, with a mean sensitivity and specificity of 58% and 72%, respectively [8]. Given that sit-to-stand transitions are key markers of functional mobility, fall risk, and disease progression, improving their reliable detection in real-world contexts is essential for enabling long-term, home-based monitoring of PD symptoms. Crucially, the proper segmentation of tasks within recorded signals is essential for developing robust algorithms that assess both the quantity and quality of mobility. Such tools are required for continuous, objective tracking of motor impairments, facilitating personalized disease management through timely adjustments in medication or rehabilitation. Ultimately, integrating multiple gross motor activities into a unified framework would enhance the potential for digital health solutions to improve symptom follow-up, mobility assessment, and treatment efficacy in everyday care of people with PD.

Accordingly, the present study revisits the previously collected dataset [8] with the goal of enhancing the detection and segmentation of *Sit* phases using a single SmartWatch worn at the ankle. Unlike the initial rule-based approach, we adopt a machine learning strategy based on random forest classification [9,10]. Random forests are particularly well-suited for this task, as they can capture complex, nonlinear relationships in sensor-derived features and have consistently demonstrated high accuracy for ADL detection using ankle-mounted sensors [11]. By leveraging this approach, we aim to advance the reliability of wearable monitoring systems and strengthen their applicability for long-term management of PD in free-living environments.

## 2. Materials and Methods

### 2.1. Participants

Twenty individuals with idiopathic PD, diagnosed according to the UK Parkinson’s Disease Society Brain Bank criteria [12], were recruited through the Quebec Parkinson Network and movement disorder specialists. Exclusion criteria included orthopaedic conditions or mild cognitive impairment (MOCA score < 18) that could interfere with task performance. One participant used a personal cane during testing. Data were collected at the Research Centre on Aging, CIUSSS Estrie–CHUS. The study was approved by the CIUSSS de l’Estrie–CHUS Institutional Review Board (MP-31-2022-4265), and all participants provided written informed consent. Patient characteristics are summarized in Table 1.

### 2.2. Experimental Procedures

The segmentation algorithm is based on the signals coming from the ankle-worn SmartWatch to minimize the impact of symptoms on ADL detection. Further details on the full protocol and the additional tests performed are available in [8], from which the dataset used here originates.

**Figure 1:**
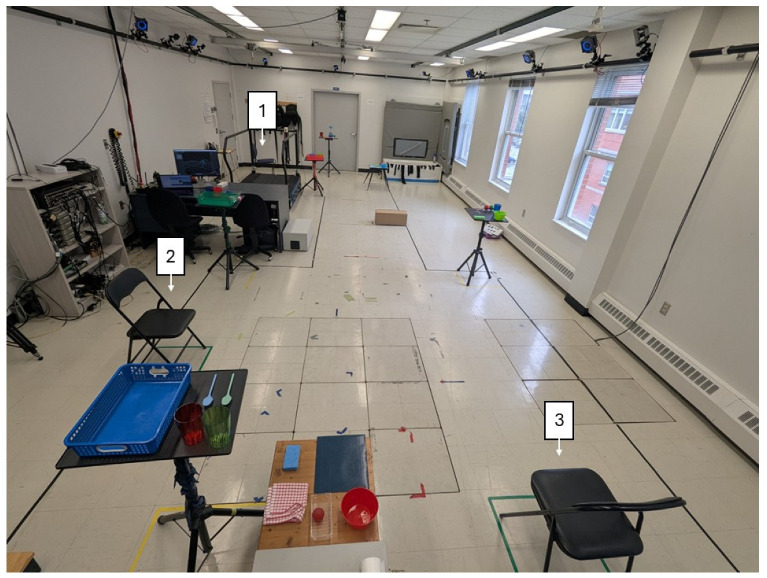
Experimental setup where participants performed the simulated free-living tasks, with the three chairs identified.

Participants performed ADLs in a simulated free-living environment during 3, 4, and 5 min trials. Color-coded objects were placed at different locations and heights, and participants collected and deposited them into matching baskets (Figure 1). Three armless chairs were available for sit-to-stand transitions. Each trial began with participants seated, after which they walked at a self-selected pace and sat in each chair at least once. Tasks were performed voluntarily to mimic natural movement, with general guidelines allowing for variability in transitions (e.g., discrete stand-to-walk vs. direct sit-to-walk). A 3 min rest was provided between trials. During testing, participants wore two commercial SmartWatches (Apple Watch Series 5, Apple Inc., Cupertino, CA, USA): one on the most affected arm and one on the contralateral ankle. Each device recorded tri-axial accelerometer and gyroscope data at 50 Hz. Note that only the SmartWatch worn at the ankle was used in the present study to develop algorithms related to the mobility of people with PD, while the SmartWatch worn at the wrist will be used in subsequent analyses to assess symptoms of patients. For reference kinematics, 39 reflective markers were tracked using 17 cameras (Optitrack, Natural Point Inc., Corvallis, OR, USA). All the measurements were collected in an On-medication state.

### 2.3. Manual Segmentation

An independent examiner (CM) manually segmented sit-to-stand and stand-to-sit transitions during the 3, 4, and 5 min trials using a full-body avatar reconstructed from OptiTrack motion capture data [8]. The examiner received standardized training and followed predefined criteria to ensure consistency. Transitions were reliably identified by marking the onset and offset of trunk and hip displacements corresponding to rising from or lowering into a seated position.

### 2.4. Data Processing and Feature Calculations

**Figure 2:**
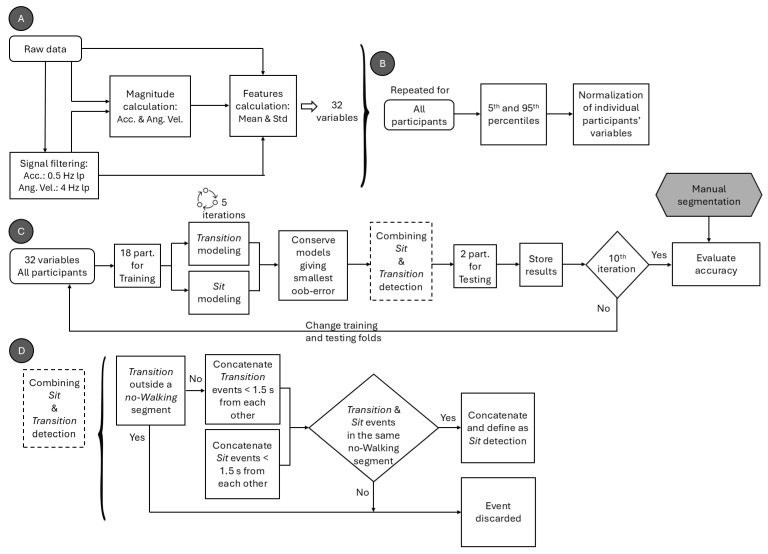
Schematic of the (**A**) data processing and variable extraction, (**B**) normalization process of the extracted variables, and (**C**) process of training and validating the classification algorithm for *Sit* phases, with (**D**) custom algorithm used to merge *Transition* and *Sit* detections. Acc. denotes acceleration; Ang. Vel. denotes angular velocity; lp denotes low pass; Std denotes standard deviation; and part. denotes participants.

The schematic of the data processing, variable extraction, and normalization process of the extracted variables is shown in Figure 2A,B. All signals were processed using second-order, zero-lag Butterworth filters. Acceleration and angular velocity magnitudes were first computed from the raw data. Low-frequency components were extracted from the tri-axial acceleration signals using a 0.5 Hz low-pass filter, and their overall magnitude was then calculated. To isolate voluntary movement, angular velocity was filtered using a 4 Hz low-pass filter, from which the filtered angular velocity magnitude was computed.

The raw and filtered acceleration and angular velocity signals were segmented in 1 s windows with 90% overlap. For each window, the mean and standard deviation of the signal were computed in the *x*, *y*, and *z* axes, as well as for the resulting magnitude of the signals. This yielded 16 features per window on the raw signal and 16 features per window on the filtered signal (Table 2). Note that the mean and standard deviation features were chosen based on the hypothesis that both amplitude and variability information of signals should discriminate between Walking and Sit phases [4]. More variables in the model would increase the complexity of the model and the risk of overfitting.

To improve the generalizability of the model, each calculated variable was normalized for each patient between the 5th and 95th percentiles, calculated on the sample of patients across all the 3 min, 4 min, and 5 min trials using Equation (1).

(1) $$Normalized\;variable\;=\;\frac{2*variable-(P5+P95)}{\left(P95-P5\right)}$$

Here, P5 and P95 correspond to the 5th and 95th percentiles, respectively. The 5th and 95th percentiles were chosen to exclude extreme low and high values that could be the result of outliers.

### 2.5. Random Forest Algorithms

**Figure 3:**
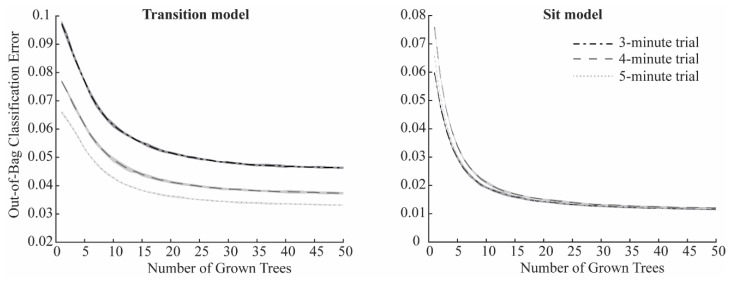
Mean out-of-bag classification error as a function of grown trees in the *Transition* model (**left**) and *Sit* model (**right**) for the 3 min trial (dash-dotted black line), the 4 min trial (dashed grey line), and the 5 min trial (dotted light grey line). Shaded areas represent the standard deviation over the 10 iterations.

Figure 2C presents the schematic of the training and validation process for *Sit* phase classification. Thirty-two features extracted from the ankle-mounted sensor were used as input to two random forest classifiers, implemented with the *TreeBagger* function in MATLAB R2023a (The MathWorks Inc., Natick, MA, USA). The first model, the *Transition* algorithm, was trained to detect stand-to-sit and sit-to-stand transitions, while the second, the *Sit* algorithm, was trained to classify the sitting state. For both models, the number of decision trees in the ensemble was optimized to balance accuracy and computational efficiency, since adding trees increases the training cost without necessarily improving performance [13]. Each algorithm was run 10 times, and mean ± SD misclassification probabilities for out-of-bag samples were calculated. Out-of-bag error curves (Figure 3) were then inspected to select an efficient ensemble size. Based on this analysis, the number of trees was set to 35 for the *Transition* algorithm and 25 for the *Sit* algorithm, which reduced training time without compromising classification performance.

After setting these parameters, the algorithms were trained and tested on the 3 min, 4 min, and 5 min trials separately with a 10-fold cross-validation, dividing each dataset into 90% training and 10% testing segments. For each fold iteration, the algorithms were trained 5 times, and the model giving the smallest out-of-bag classification error was used for testing.

**Figure 4:**
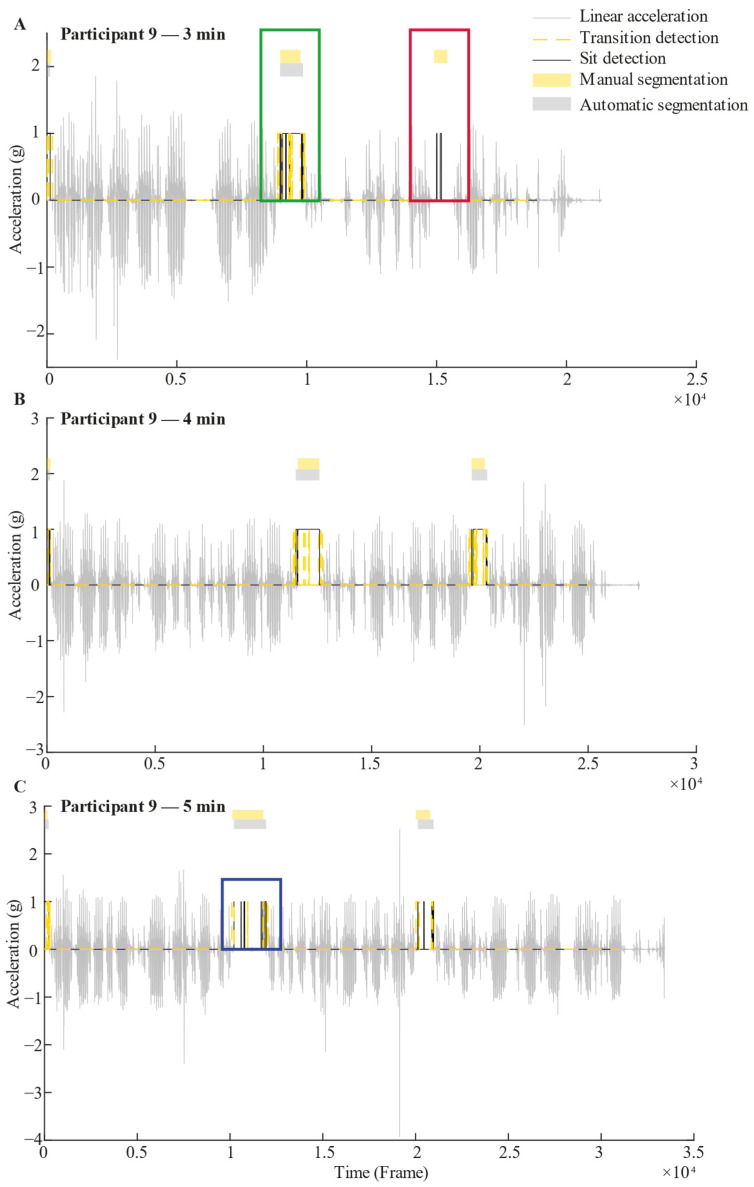
Example of 3 min (**A**), 4 min (**B**), and 5 min (**C**) recorded signals with their respective segmentation of Sit phases for one participant. The grey signal represents linear vertical acceleration. The black line is a representation of the Sit detection output, while the dashed yellow line is a representation of the Transition detection output, based on random forest algorithms. These black and yellow lines take a value of 1 when a Sit or Transition is detected, and 0 otherwise. Above the signal, the yellow rectangles represent Sit phases that were manually segmented by an independent examiner. Just below, the grey rectangles indicate Sit phases that were detected by the algorithm by combining results of the Transition and Sit detection outputs; i.e., for a Sit phase to be validated, both Sit and Transition detections must occur within the same no-Walking segment, as illustrated in the green square (Panel **A**). In contrast, if a Sit detection is not accompanied by a Transition detection within the same no-Walking segment, the Sit phase is considered invalid, as shown in the red square (Panel **A**). When multiple Sit detections occur within a single no-Walking segment, they are concatenated to form a single Sit phase, as demonstrated in the blue square (Panel **C**).

In a subsequent step, a custom algorithm (Figure 2D) was implemented to merge *Transition* and *Sit* detections to classify the *Sit* phases. For this, all *Transition* or *Sit* detections separated by less than 1.5 s were concatenated based empirically on the dataset. *Sit* detections that were not associated with a *Transition* detection in the same *no-Walking* segment were discarded. Note that *Walking* and *no-Walking* segments were automatically detected using a previously validated algorithm [8]. Also, *Transition* detections outside a *no-Walking* segment were discarded. *Sit* segments detected in the same *no-Walking* segment were concatenated. Figure 4 shows examples of 3 min, 4 min, and 5 min segmentation for one participant.

### 2.6. Statistical Analyses

Sensitivity, specificity, and F-score were calculated to evaluate *Sit* phase detection performance across the 3, 4, and 5 min trials. Segmentation accuracy was assessed by comparing the manually annotated start and end times of each Sit segment with those identified by the algorithm. Temporal precision was quantified as the absolute difference between manually and algorithm-detected time points (ΔT = |T_manual − T_algo|). The boxplot of the ΔT was generated for all the 3 min, 4 min, and 5 min trials to show the predictive reliability of using the algorithm to segment *Sit* phases.

## 3. Results

### 3.1. Sit Phase Detection

The datasets were composed of 60 events, 66 events, and 65 events of *Sit* phases for the 3 min, 4 min, and 5 min trial, respectively. Table 3 summarize the *Sit* phase detection results of the proposed algorithm for the 3 min, 4 min, and 5 min trials.

### 3.2. Activity Segmentation

**Figure 5:**
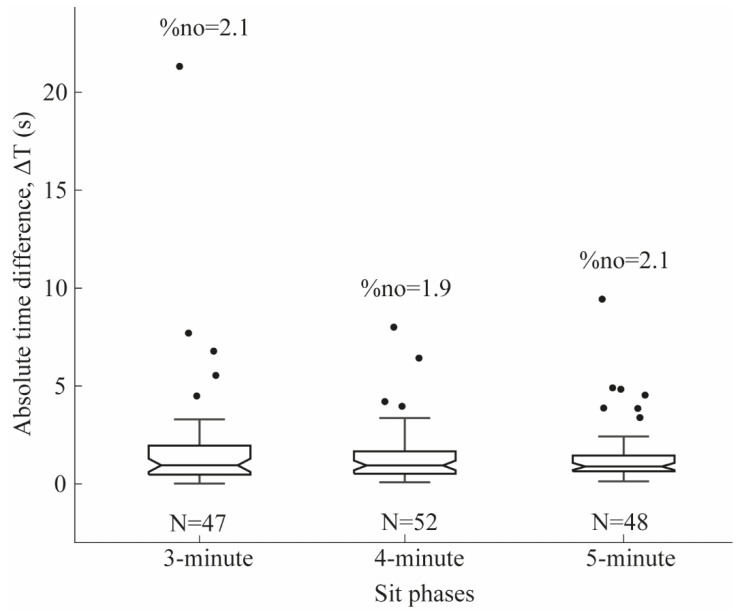
Absolute time difference (ΔT) representing the temporal precision between manual and automatic segmentations. N denotes the number of task events for the 3 min, 4 min, and 5 min trials, and %no indicates the percentage of outliers within the 3 min, 4 min, and 5 min trials.

Figure 5 shows the absolute time difference (ΔT) between manual and automatic segmentations. For the 3 min trial, the median ΔT between the manual and automatic segmentation of *Sit* phase events (N = 47) was 0.95 s, with five outliers (%n_o_ = 2.1).

For the 4 min trial, the median ΔT between the manual and automatic segmentation of *Sit* phase events (N = 52) was 0.89 s, with four outliers (%n_o_ = 1.9).

For the 5 min trial, the median ΔT between the manual and automatic segmentation of *Sit* phase events (N = 48) was 0.84 s, with seven outliers (%n_o_ = 2.1).

## 4. Discussion

This study examined the detection and segmentation of *Sit* phases in people with PD using a single ankle-worn SmartWatch. Random forest classifiers were employed, achieving high sensitivity and specificity for event detection and segmentation during simulated free-living tasks.

In a previous study [8] on detecting and segmenting gross motor activities during ADLs in people with PD, *Sitting down* and *Standing up* were identified with only moderate precision (F-scores of 52.3% and 54.1%). These results underscore the need for improved methods that are capable of achieving higher detection accuracy for these tasks in PD.

Sun et al. [4] reported 94% precision for *Sit* phase detection using a custom wrist-based algorithm that relied on the mean and variance of acceleration signals. However, their recordings were task-specific rather than continuous, preventing interference from other activities as in the present study, where participants performed multiple ADLs freely during 3, 4, and 5 min trials. Moreover, Sun et al. [4] did not report participants’ demographic characteristics, limiting the generalizability of their findings to populations with PD, where reduced arm swing significantly affects wrist-based detection [5].

Other ankle-based studies have shown more modest performance. Tang et al. [14] achieved an F-score of 81% for *Sitting* detection using a support vector machine classifier with data from young healthy adults, but no validation was conducted in people with PD. Davoudi et al. [15] applied a random forest algorithm with ankle-worn accelerometers (Actigraph GT3X) and reported 65% accuracy for sedentary activity detection in healthy older adults.

In contrast, the present study applied random forest classifiers to first detect sit-to-stand and stand-to-sit *Transitions*, followed by *Sit* segments. The combined algorithm achieved high precision for *Sit* phase detection during simulated free-living tasks, with F-scores of 84.7%, 80.6%, and 75.6% for the 3, 4, and 5 min trials, respectively. Furthermore, the median time difference between manual and automatic segmentations averaged 0.89 s, confirming that *Sit* phases in people with PD can be detected and segmented with high temporal precision using a single ankle-worn SmartWatch.

Previous studies have explored various approaches to detect sit-to-stand and stand-to-sit *Transitions* and *Sit* phases in both healthy individuals and people with PD, using wrist-worn SmartWatches [16], waist- or lower-back-mounted inertial sensors [17,18], smart insoles [19], and even video-based systems [20]. To the best of our knowledge, the present study is the first to demonstrate high accuracy in detecting and segmenting *Sit* phases in people with PD using only a single ankle-worn SmartWatch. This finding expands the possibilities for practical, wearable-based monitoring of functional mobility in PD.

Importantly, by leveraging random forest classification algorithms [9,10], our method offers greater generalizability and reduces the risk of misclassification compared with simpler approaches, such as decision trees or threshold-based rules [4,8]. This robustness is particularly relevant for long-term monitoring of mobility and symptoms in real-life contexts [21,22]. Nonetheless, further validation in true free-living environments and across new patient cohorts is required to confirm the reliability and scalability of these algorithms under real-world conditions.

A limitation of this study comes from the developed algorithm. Since we chose to segment the raw and filtered acceleration and angular velocity signals in 1s windows before calculating the mean and standard deviation that were used as input variables of the random forest algorithms, it is possible that Sit events lasting less than 1s could be undetectable. However, we believe that missing Sit events lasting less than 1s could be considered clinically acceptable for the purpose of mobility assessment, since such short Sit phases would not affect the estimation of the sedentary and active phases. Another limitation is that participants had relatively mild disease, with a mean Hoehn and Yahr score of 1.4 and a maximum of 2. Future work should validate the algorithm in patients at more advanced stages of PD. Nonetheless, when combined with our previously validated *Walking* and *Turning* algorithms [8], the present findings represent a substantial advancement in detecting and segmenting gross motor activities during ADLs using a single ankle-worn SmartWatch—an important step toward reliable home-based monitoring in PD. Future experiments should now be conducted in real home and real living environments instead of a simulated free-living environment to better reflect the randomness of varied activities and environments, as well as to validate these algorithms in ecological settings.

## 5. Conclusions

Taken together with our previous findings [8], the present results demonstrate that relatively simple algorithms, when combined with random forest classifiers applied to inertial data from a single ankle-worn SmartWatch, can accurately detect and segment core gross motor ADLs in people with PD, including walking, turning, and sit phases. By capturing these fundamental movements with high temporal precision, the approach provides an ecologically valid means of evaluating patients’ mobility quality in their natural living environments using only one unobtrusive sensor. This advancement carries important clinical implications. It enables continuous, objective monitoring of mobility impairments with a simple single-sensor setup, enhancing feasibility for long-term home use. It also supports more personalized disease management, where motor fluctuations can guide timely medication or rehabilitation adjustments. Moreover, integrating multiple gross motor activities into one framework moves toward scalable digital health solutions for everyday PD management.

## Figures and Tables

**Table 1:** Characteristics of patients.

Characteristics	Mean ± SD	Range
***Patients (n = 20, 10 Females)***		
Age (year)	66.6 ± 9.0	47–78
Weight (kg)	74.3 ± 13.4	50–97
Height (cm)	166.9 ± 8.9	152–180
Years since diagnosis *	7.0 ± 5.5	1–21
Comorbidity index (/18)	5.1 ± 2.4	1–9
Moca (/30)	27.3 ± 2.8	19–30
Notthingham ADL scale (/22)	19.7 ± 1.7	16–22
***MDS-UPDRS Part III*** (*On* state) **		
Speech (3.1)	0.4 ± 0.6	0–2
Facial expression (3.2)	0.6 ± 1.0	0–4
Neck rigidity (3.3)	0.7 ± 0.8	0–2
Arm rigidity (3.3)	0.9 ± 0.8	0–2
Leg rigidity (3.3)	0.7 ± 0.7	0–2
Finger tapping (3.4)	0.6 ± 0.7	0–2
Hand movements (3.5)	0.8 ± 0.6	0–2
Pro-sup movements of hands (3.6)	0.7 ± 0.7	0–2
Toe tapping (3.7)	0.3 ± 0.4	0–1
Leg agility (3.8)	0.3 ± 0.6	0–2
Arising from chair (3.9)	0.1 ± 0.3	0–1
Gait (3.10)	0.4 ± 0.8	0–2
Freezing of gait (3.11)	0.1 ± 0.2	0–1
Postural stability (3.12)	0.7 ± 0.8	0–2
Posture (3.13)	0.4 ± 0.5	0–1
Body bradykinesia (3.14)	0.3 ± 0.5	0–1
Postural tremor (3.15)	0.4 ± 0.6	0–2
Kinetic tremor (3.16)	0.7 ± 0.6	0–2
Rest tremor amplitude upper limbs (3.17)	0.6 ± 0.8	0–3
Constancy of rest tremor (3.18)	1.4 ± 1.3	0–4
Hoehn and Yahr score On	1.4 ± 0.5	1–2

* One participant with missing information. ** Two participants with missing information.

**Table 2:** Type and number of variables calculated for each feature.

Variable	N Variables Acceleration	N Variables Ang. Velocity
Mean	8	8
Standard deviation	8	8
		
Ankle	16	16

Std denotes standard deviation.

**Table 3:** Sensitivity, specificity, and F-score of *Sit* phase detection for the 3 min, 4 min, and 5 min trials.

Trial	Sensitivity (%)	Specificity (%)	F-Score
3 min	78.3	93.8	84.7
4 min	78.8	85.5	80.6
5 min	71.6	84.8	75.6

## Data Availability

The aggregated data presented in this study are available on request from the corresponding author due to ethical constraints.

## Abbreviations

The following abbreviations are used in this manuscript:

ADLs	Activities of daily living
PD	Parkinson’s disease
